# Assessment of accuracy and efficiency of guided fixation of customized 3D miniplates vs Champy’s technique in stabilization of mandibular angle fractures: A randomized controlled trial

**DOI:** 10.1007/s00784-026-07118-w

**Published:** 2026-10-02

**Authors:** Arwa Hosni, Mohamed Shawky, Mohamed Ibrahim Sakr, Waleed El-Beialy, Mostafa Ezz, Mohammed Atef

**Affiliations:** 1 Department of Oral and Maxillofacial Surgery, Faculty of Oral and Dental Medicine, Future University in Egypt, PO Box 11835, Cairo, Egypt; 2 Department of Oral and Maxillofacial Surgery, Faculty of Dentistry, Cairo University, PO Box 11553, Cairo, Egypt

**Keywords:** Mandibular angle fractures, Virtual surgical planning, Patient-specific devices, Bite force, Customized 3D miniplate

## Abstract

**Objectives:**

The objectives of this study were; (1) to assess the accuracy of virtual surgical planning (VSP) and computer aided design and manufacturing (CAD/CAM) in creating an anatomically oriented, bone-borne fracture reduction/plate positioning patient specific surgical guide (PSSG) and a customized three-dimensional (3D) miniplate to achieve anatomical reduction and stabilization of fractured segments in mandibular angle fractures (MAF) via a more esthetic, less complication-laden intraoral access, and (2) to evaluate efficiency of customized 3D miniplates in reestablishment of masticatory function.

**Methods:**

Twenty-four patients were included and randomly allocated into 2 groups; *group I*, where open reduction and internal fixation (ORIF) of MAF was performed using a fracture reduction/plate positioning PSSG and a customized 3D miniplate and *group II,* where ORIF of MAF was performed using a single 2.0 superior border miniplate (Champy’s technique). Assessment of accuracy of VSP in *group I* was carried out by superimposition of 3D virtual models of virtually preoperatively reduced mandible over those of surgically reduced and stabilized mandibles at 1 week and 6 months postoperatively. Clinical assessments of maximum bite force in Newtons (N) using a bite force measurement device as well as occlusion status according to the following scale: satisfactory (no gap), mild derangement (gap of 1–2 mm) or marked derangement (gap more than 2 mm) were carried out at 1 week, 1, 3 and 6 months postoperatively.

**Results:**

In *group I*, superimposition of 3D virtual models of surgically reduced and stabilized mandibles at 1 week and 6 months over the virtually preoperatively reduced mandible revealed a mean surface deviation of 0.36 ± 0.09 mm and 0.45 ± 0.08 mm, respectively. Bite force measurements in *group I* were significantly higher (p < 0.001) than those in *group II* at 1 week (20.48 ± 3.20N—13.24 ± 2.83N), 1 month (69.52 ± 3.42N—41.16 ± 3.02N) and 3 months (147.45 ± 3.00N—139.50 ± 3.44N), respectively, with large effect sizes (PES > 0.29). At six months, there was also a statistically significant difference (p = 0.003) between both groups (166.66 ± 2.8N—162.31 ± 3.19N respectively) with higher bite force in group I, however, the effect size was moderate (PES = 0.13). Difference in postoperative occlusion status was statistically insignificant between both groups across all time points.

**Conclusion:**

Assessment of accuracy of VSP revealed a minimal amount of discrepancy between 3D virtual models of surgically reduced and stabilized mandibles over those of virtually preoperatively reduced mandibles. An anatomically oriented, bone-borne fracture reduction/plate positioning PSSG aided significantly in customized 3D miniplate positioning and fracture reduction. Fractures stabilized with a customized 3D miniplate were associated with higher bite forces when compared to single miniplate fixation, however, this must be interpreted with caution. We recommend that future studies be conducted on a larger sample size.

Clinical relevance.

Based on the results of this study, we believe that utilizing VSP and CAD/CAM for guided ORIF of MAF using customized 3D miniplates seems to be a promising method, allowing for predictable 3D miniplate positioning and fixation through an intraoral approach and may allow for early reestablishment of masticatory function.

Clinical trials registration.

This study was registered on ClinicalTrials.gov (ID: NCT06392204), verification date April 2024. https://clinicaltrials.gov/study/NCT06392204

## Introduction

The human mandible, due to its prominence, is frequently involved in fractures of the facial bones. Among mandibular fractures, the incidence of mandibular angle fractures (MAFs) is relatively high (27%–30%)[1]. Over the years, management of MAFs was rather controversial as these are complicated by the thin cross-section of bone in this area, intricate biomechanical relations, the presence of the third molar and a high rate of postsurgical complications [2–6]. Despite the controversy over the best surgical management protocol for MAFs, the primary objectives remain agreed upon; to achieve as close an anatomical reduction as possible to ensure bone healing in that position, followed by fixation and stabilization of the fractured segments in order to restore functional occlusion and masticatory efficiency as well as reinstatement of a proper condylar position[7]. The literature reports a variety of techniques for managing MAFs, including closed reduction with maxillomandibular fixation (MMF) and open reduction and internal fixation (ORIF) with either; transosseous wiring, lag screws or plate osteosynthesis via an intraoral or extraoral approach [1, 8–10].

Consensus is believed to have been reached in the fact that ORIF is the gold standard for management of MAFs since closed reduction with MMF does not result in the stability of the proximal fragment leading to anterosuperior rotation [11]. Intraoperatively, reduction of mandibular fractures is generally achieved by using either; MMF, reduction forceps, manual reduction, or a combination of these methods. Precise anatomical reduction of the fractured segments in MAFs with some of the aforementioned techniques has proven clinically difficult, with possible resultant postoperative complications such as malunion and nonunion leading to; postoperative infection, malocclusion, improper condylar position, as well as being time consuming [12, 13]. Of the most widely accepted and used plate osteosynthesis methods to date is Champy’s technique, which is believed to establish a functionally stable fixation using a single miniplate fixed along the external oblique ridge via a conservative intraoral approach, nevertheless, some studies have questioned Champy’s stress distribution principles for fracture stabilization based on biomechanical findings, stating reversal of tension and compression zones when load is applied posteriorly in proximity to the MAF resulting in inferior border gapping of the fracture line which is suspected to contribute to the occurrence of postoperative complications [5, 14]. Consequently, surgeons began opting towards the use of two miniplates (one fixed at the superior border intraorally and the other at the inferior border of the mandible transbucally) or a rigid load-bearing plate at the inferior border [15, 16]. Nevertheless, inferior border plating transbucally has been reported to be technique sensitive [16],while rigid load-bearing plate fixation necessitates the need for an extraoral approach for proper plate adaptation and fixation with consequent scarring, possible marginal mandibular nerve damage, and increased operating time [10, 11].Farmand was the first to introduce three- dimensional (3D) miniplates, they’re essentially 2 miniplates joined by vertical bars designed to counteract torsional forces along the long axis of the miniplates thus increasing stability of the fixed fractured segments when subjected to occlusal load, allowing earlier reestablishment of masticatory function [17]. Masticatory function refers to the ability to chew without any interference or pain. Its major determinants are the range of mandibular motion, maximum occlusal forces, and the activity of the masticatory muscles, therefore, bite force measurements are an excellent criterion for the assessment of masticatory efficiency[18]

With the rising pace of technology worldwide, virtual surgical planning (VSP) and customized patient specific surgical devices (PSSD) based on computer aided design and manufacturing (CAD/CAM) platforms are now at the frontier of the medical field. PSSDs can be broadly classified into five categories, based on their use in Oral and Maxillofacial Surgery; 3D anatomic models, surgical guides (cutting/drilling/positioning), occlusal splints, patient specific implants (PSI) such as osteosynthesis plates, skeletal reconstruction devices, and facial prostheses[19]. Patient specific plates were supported by many studies for use in craniomaxillofacial reconstruction[20, 21]. Patient specific osteotomy guides were also used in orthognathic surgeries[22], drill guides were also incorporated in those CAD/CAM patient specific guides [23]. Such predesigned devices provide high accuracy with precise adaptation to bone, less intraoperative time, less tissue morbidity and reduced surgical complications which are important factors that influence the surgical outcome [24].

The aim of this study was to assess the accuracy of VSP and CAD/CAM in creating an anatomically oriented, bone-borne fracture reduction/plate positioning patient specific surgical guide (PSSG) and a customized 3D miniplate to achieve anatomical reduction and stabilization of fractured segments in MAF via a more esthetic, less complication-laden intraoral access, and to evaluate efficiency of customized 3D miniplates in reestablishment of masticatory function.

### Materials and methods

This study was approved by the Research Ethics Committee of the Faculty of Dentistry at Cairo University (2–4–23) and registered on ClinicalTrials.gov (ID: NCT06392204). The study adopted a single-center, single-blinded, randomized controlled trial design and followed the Consolidated Standards of Reporting Trials Statement. A power analysis was designed to have adequate power to apply a two-sided statistical test of the null hypothesis that there is no difference between different tested groups regarding post-reduction fracture gap. By adopting an alpha (α) level of (0.05), a beta (β) level of (0.2) (i.e. power = 80%) and an effect size (d) of (1.5) calculated based on the results of a previous study by Khandeparker et al.[25] which compared management of mandibular angle fractures using a single miniplate via transoral versus transbuccal approaches; **t**he predicted sample size (n) was a total of (16) cases (i.e. 8 cases per group). Sample size was increased by (25%) to compensate for possible drop-out during different follow-up intervals to be a total of (20) cases (i.e. 10 cases per group). Sample size calculation was performed using G*Power version 3.1.9.7[26].

Twenty-four patients were included in this study, from the selected eligible patient population at the Department of Oral and Maxillofacial Surgery, Faculty of Dentistry, Cairo University. Inclusion criteria entailed the presence of unilateral MAF -with or without other mandibular fractures- requiring ORIF in patients above 18 years old with sufficient dentition to reproduce the occlusal relationships. Patients with comminuted mandibular angle fracture(s) and angle fracture with intra-bony lesions that may interfere with proper anatomical reduction and those undergoing chemotherapy or radiotherapy related to the head and neck region or suffering from any systemic diseases that might retard normal healing were excluded. All included patients signed a consent form to participate in the study and were allocated into 2 groups; *group I*, where ORIF of the MAF was guided by an anatomically oriented, bone-borne fracture reduction/plate positioning PSSG and fixed with a customized 3D miniplate and *group II*, where ORIF of MAF was performed using Champy's technique. Participants were randomly allocated using a block randomization method. The randomization sequence was generated using a computer-generated list with fixed block sizes to ensure balanced group distribution. Allocation concealment was achieved using sealed opaque envelopes. A thorough preoperative assessment of all patients was carried out including collection of a complete medical history and thorough clinical examination. A panoramic radiograph was acquired for all included patients to confirm the presence of MAF. All surgical procedures were performed under general anesthesia.

**Fig. 1:**
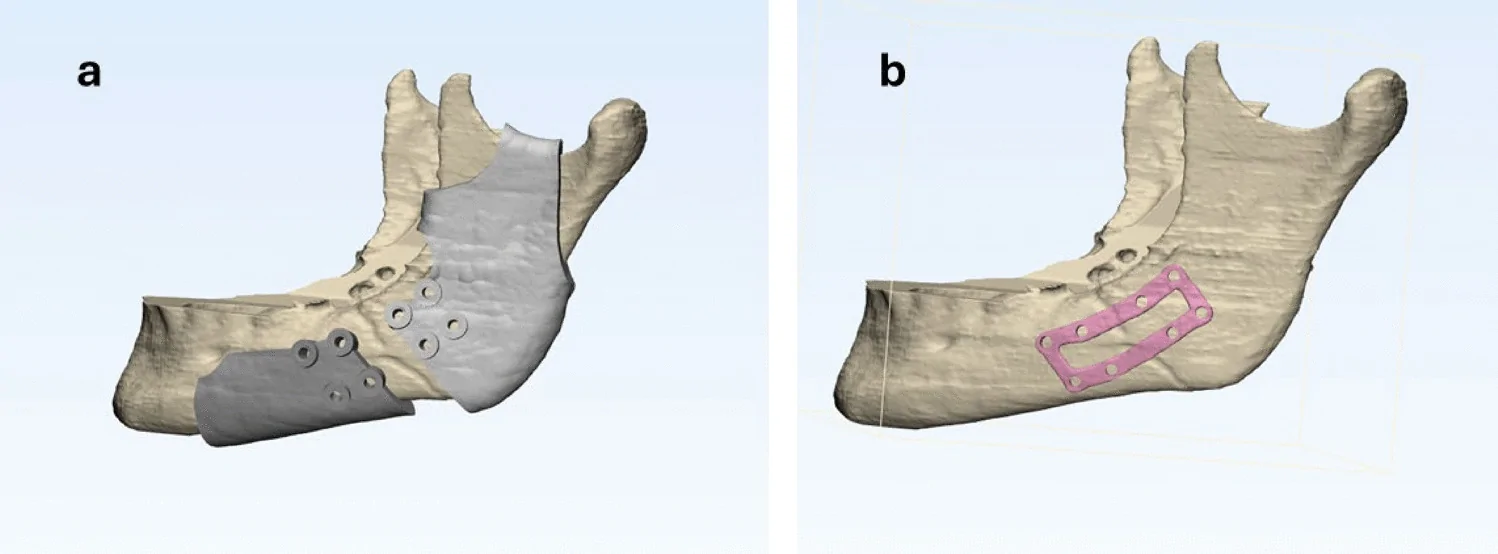
A virtual 3D model of the reduced mandible by VSP; **a**) with the bone-borne fracture reduction/plate positioning PSSG, and **b**) with the customized 3D miniplate

**Fig. 2:**
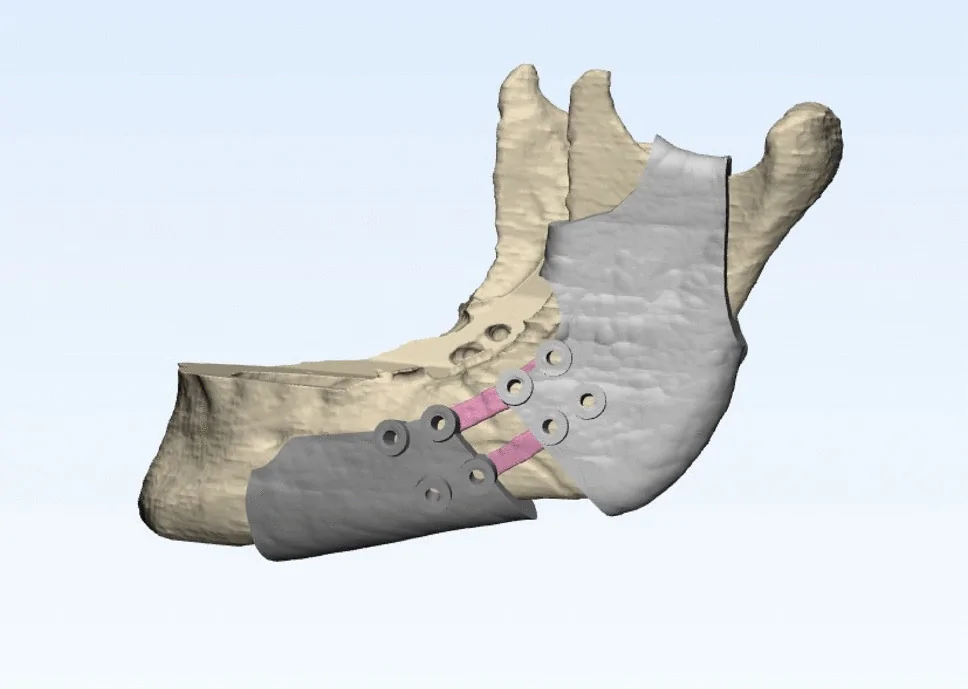
A virtual 3D model of the reduced mandible by VSP showing the fracture reduction/plate positioning PSSG drill guiding holes coinciding with the screw holes of the customized 3D miniplate

**Fig. 3:**
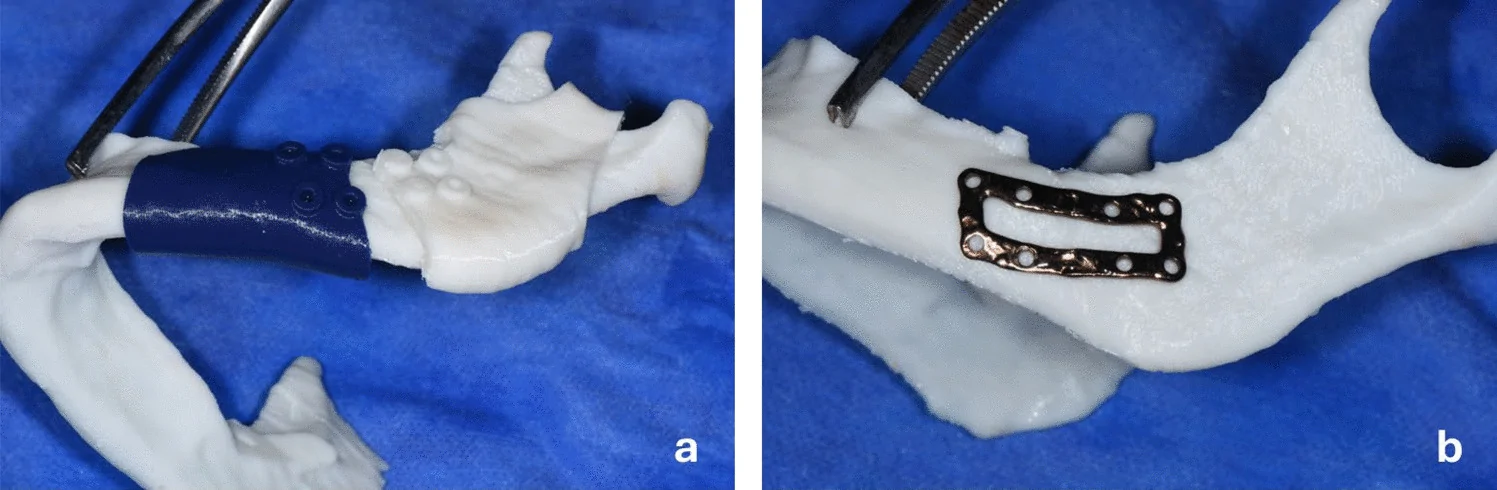
A 3D printed model of the virtually reduced mandible: **a**) showing the adaptation of the bone-borne 3D printed fracture reduction/plate positioning PSSG and **b**) showing the passive adaptation of the customized 3D miniplate

For patients allocated to group I, a Computed tomography (CT) scan of the head and neck region was also acquired, followed by:

- (a) Virtual surgical planning and computer aided design of fracture reduction/plate positioning surgical guides and 3D miniplates: For each patient, digital imaging and communication in medicine (DICOM) files of their preoperative CT scans were imported to a specialized viewer and planning software (Mimics21, Materialise, Leuven, Belgium) to obtain axial, coronal and sagittal views, followed by segmentation using thresholding to produce a mask for the entire patient’s skull then a separate mask for the mandible was created using the split mask tool. Each fracture segment of the mandible was then obtained as a separate mask. Virtual 3D models were then calculated for the obtained masks of the mandible. The virtual 3D models were then imported into a second software (3-Matic, Leuven, Belgium) specialized in designing medical devices and surgical stents. The virtual 3D models of the fractured mandibular segments were virtually reduced anatomically and managed as a single entity, after which the two-piece fracture reduction/plate positioning PSSG with drill guiding holes was designed. The distal part of the guide was designed to slightly engage the inferior border of the mandible and the proximal part was designed to slightly engage the sigmoid notch, the posterior border, the inferior border and angle of the mandible at the site of fracture (Fig. 1a). Design considerations included allowing visualization of the fracture line intraoperatively and avoiding injury to the inferior alveolar canal and roots of teeth during plate fixation. Superiorly, both pieces of the guide were designed to be positioned clear off any gingival soft tissue. A customized 3D miniplate was similarly designed for fixation and stabilization of the fractured segments; the plate profile was set to 1 mm thickness (Fig. 1b). The drill guiding holes of the surgical guide were designed to coincide with the screw holes of the 3D miniplate (Fig. 2).
- (b) Construction of the reduction/plate positioning surgical guides and 3D miniplates via computer aided manufacturing: The two-piece PSSG was 3D printed in polylactic acid filament (PLA +, eSun, Shenzhen Esun Industrial Co., Ltd, China) using a 3D printer (Prusa MK3S, Prusa Research a.s., Czech Republic), they were then cold sterilized preoperatively by immersion in 2% glutaraldehyde solution for 12 hours followed by sterile 0.9% saline wash (Fig. 3a). The 3D miniplates were milled from Grade 5 titanium alloy using a CAD/CAM milling machine (ED5X, EMAR, 10th of Ramadan City Asharqia, Egypt) then properly debrided, washed and sterilized (Fig. 3b).

### Surgical procedure for Group I

**Fig. 4:**
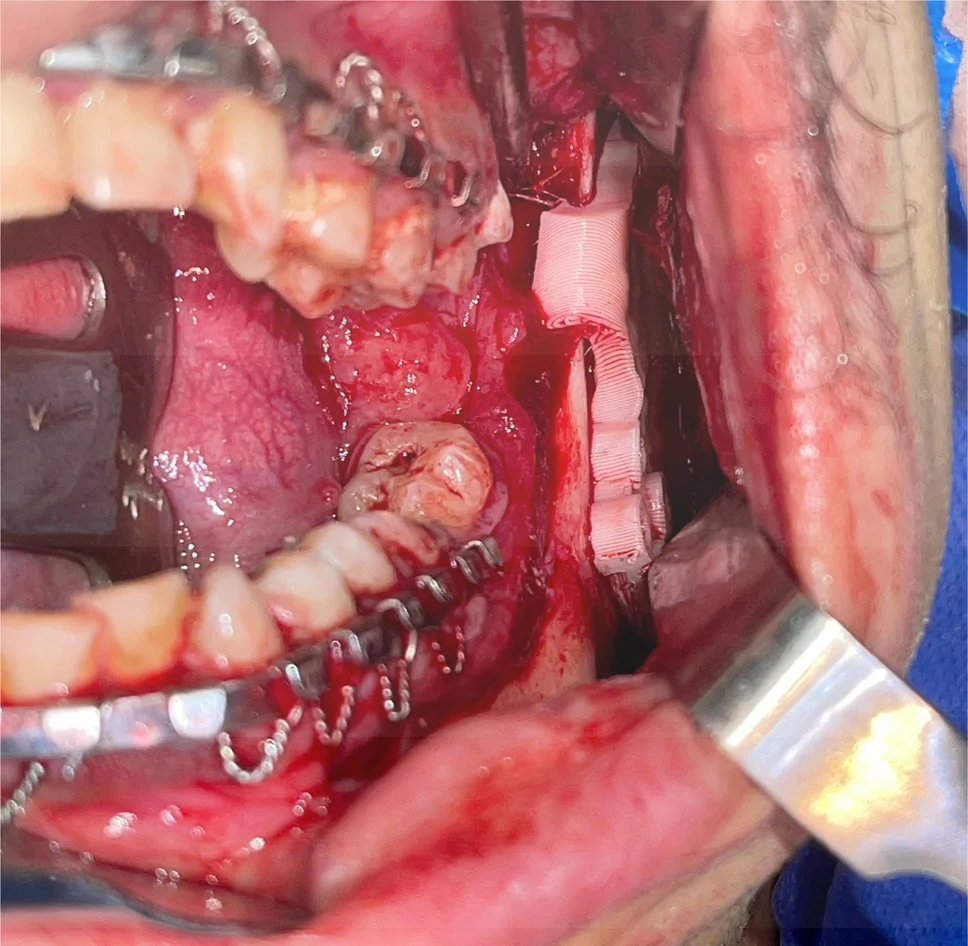
An intraoperative photograph showing the fit of the proximal segment of the fracture reduction/plate positioning PSSG in the predestined position according to the virtual plan

**Fig. 5:**
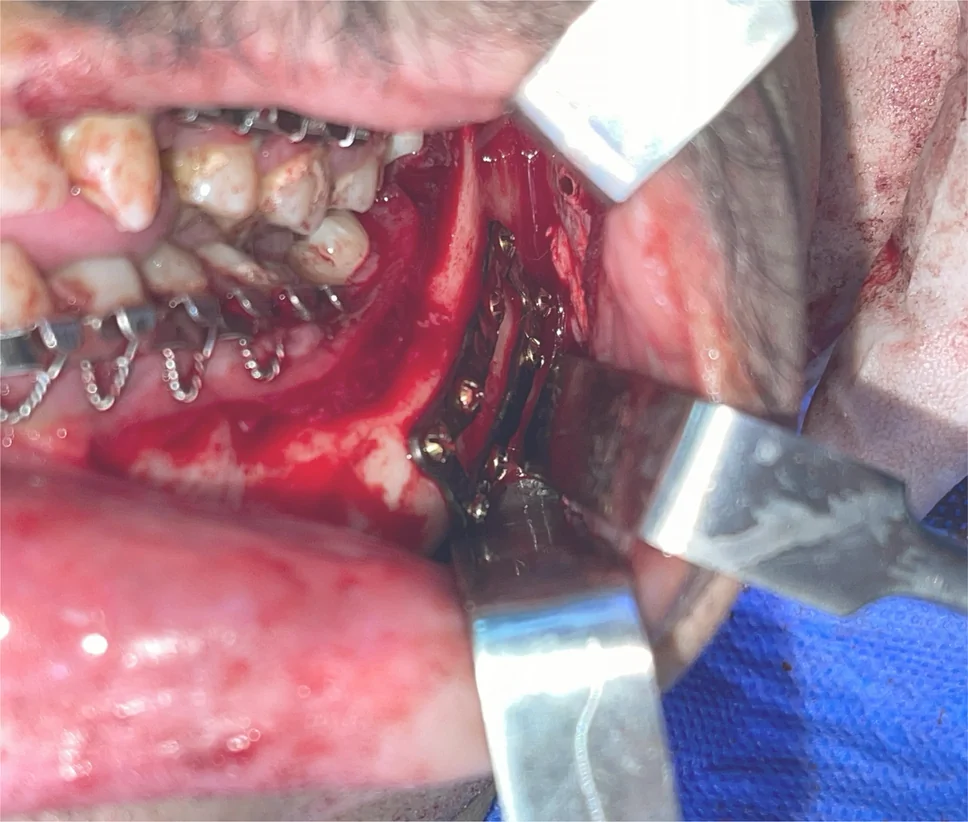
An intraoperative photograph showing the customized 3D miniplate fixed with 2.0 screws at the predestined position according to the virtual plan

Scrubbing of the surgical site and draping was carried out in a standard protocol using betadine surgical scrub followed by injection of local anesthesia intraorally, after which an Obwegeser incision was done using a bard-parker blade no. 15 and a full thickness mucoperiosteal flap was reflected to expose the buccal and lingual aspects of the ramus- angle region up to the coronoid notch superiorly. A J-stripper was then used for ptreygo-masseteric muscle sling release to expose the most posteroinferior border of the mandible, the angle region and the posterior border of the ramus at the site of fracture. The fracture line was then identified and debrided from intervening fibrous tissue followed by MMF to obtain centric occlusion. After properly obtaining centric occlusion, the proximal segment of the PSSG was placed in the predestined position decided in the virtual plan (Fig. 4). Monocortical drilling was then carried out through the drill guiding holes coinciding with the plate screw holes using a transbuccal trocar via a stab extraoral incision or transorally when convenient. The proximal segment of the PSSG was then removed, and the distal segment was placed. Monocortical drilling was performed in a similar fashion. The customized 3D miniplate was then passively placed and fixed at the predrilled screw sites with 2.0 screws using a transbuccal trocar (Fig. 5). Debridement and irrigation of the surgical field with saline was carried out followed by intra-oral incision closure with locked continuous suturing using 3–0 vicryl sutures. Extra-oral stab incision was sutured with monofilament polypropylene non-resorbable sutures.

### Surgical procedure for Group II

**Fig. 6:**
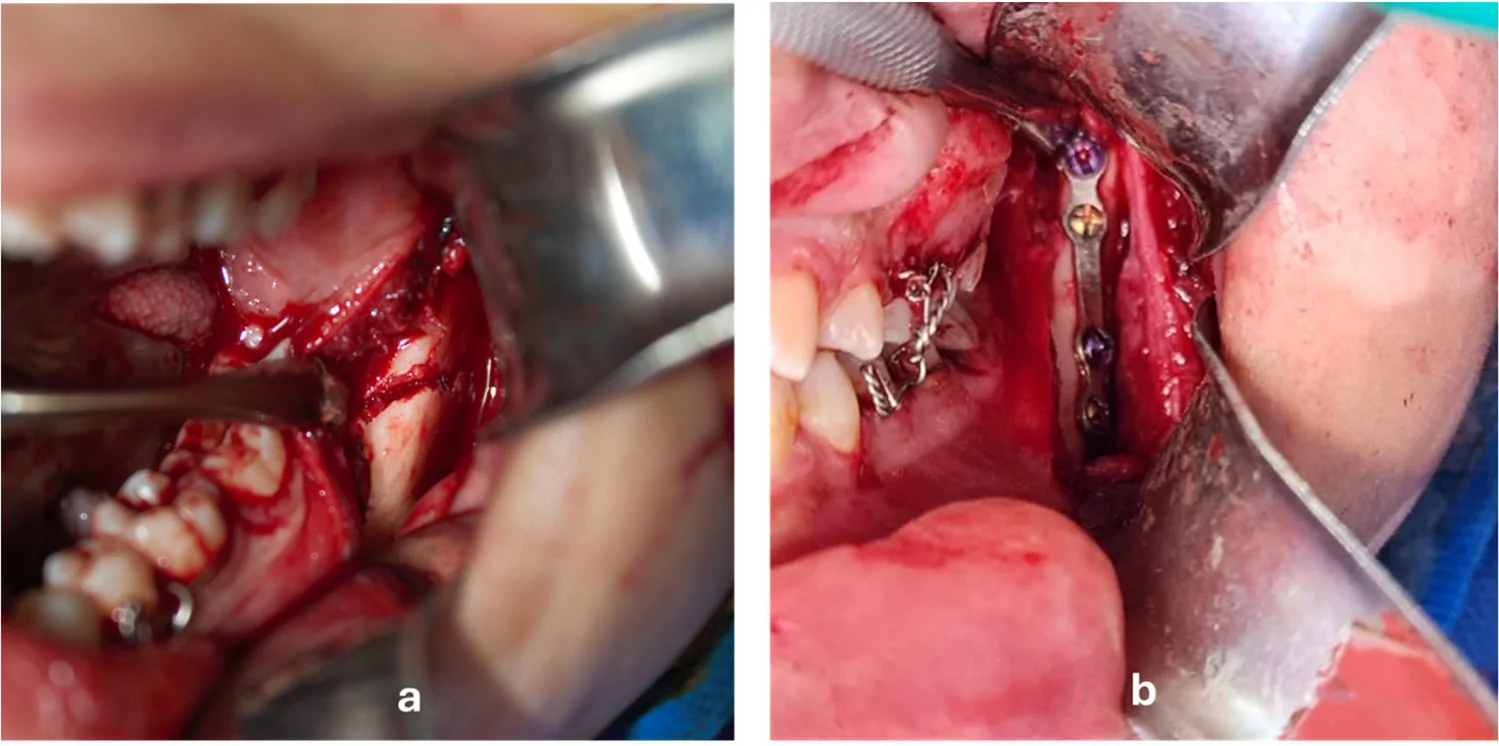
Intraoperative photographs showing: a) a left mandibular angle fracture through an Obwegeser incision and b) fractured segments reduced and fixed with 2.0 miniplate using Champy’s technique

Scrubbing, draping and injection of local anesthesia were performed as in *group I*, an Obwegeser incision was performed using a bard-parker blade no. 15 to expose the buccal and lingual aspects of the ramus angle region. The fracture line was then identified and debrided from intervening fibrous tissue (Fig. 6a). MMF was performed to obtain centric occlusion. Fractured segments were then manually reduced and a 2.0 miniplate was bent, fitted and screwed to the superior border of the mandible at the area of the external oblique ridge (Fig. 6b). MMF was then released, and occlusion checked. Debridement and irrigation of the surgical field was carried out followed by intra-oral incision closure with locked continuous suturing using 3–0 vicryl sutures.

**Fig. 7:**
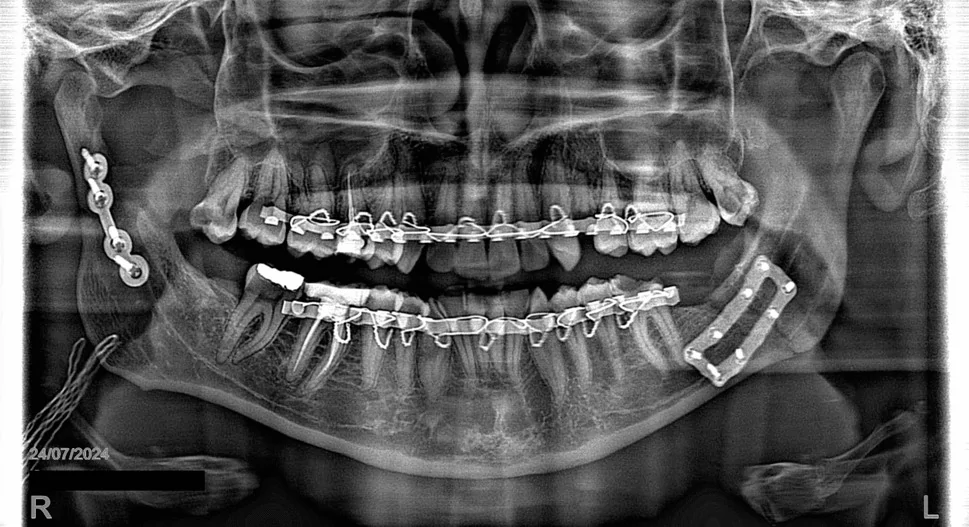
A panoramic radiograph at 1 week postoperative showing fixation of a left mandibular angle fracture using a customized 3D miniplate

All patients were instructed to apply cold compresses in the form of ice packs for 20 minutes every hour for the first 24 hours postoperatively to minimize edema, maintain a soft diet and were kept on the following medications regimen: Oral antibiotic (Amoxicillin/Clavulanic acid 1000 mg)^1^ every 12 hours for 5–7 days postoperatively, Oral Metronidazole^2^ 500 mg every 8 hours for 5 days postoperatively, Ibuprofen^3^ 600 mg every 8 hours for 3- 5 days postoperatively, and Chlorhexidine^4^ mouthwash twice daily for 10 days. Postoperative panoramic radiographs were obtained for all patients at 1 week (Fig. 7) and 6 months postoperatively. CT scans were obtained for *group I* at 1 week and 6 months postoperatively.

Clinical assessments of bite force and occlusion status were carried out at follow-up intervals of 1 week, 1 month, 3 months and 6 months postoperative. Occlusal discrepancy was assessed based on a scale used in previous studies by Mujtaba et al.[8] and Vineeth et al. [27], using a millimeter caliper to measure the distance between the upper and lower first molars at maximum intercuspation and was categorized as satisfactory (no gap), mildly deranged (1–2 mm gap), and deranged (> 2 mm gap).

Assessment of bite force in Newtons (N) was performed for all patients using a bite force measurement device (BYTE, Innovatios technology, Bengaluru, Karnataka, India) to measure maximum bite force in Newton scale in three areas, right first molar area, left first molar area and central incisors area with the head in an upright unsupported natural position, similar measurements were performed for a group of 12 healthy controls for purposes of device calibration and comparison.

**Fig. 8:**
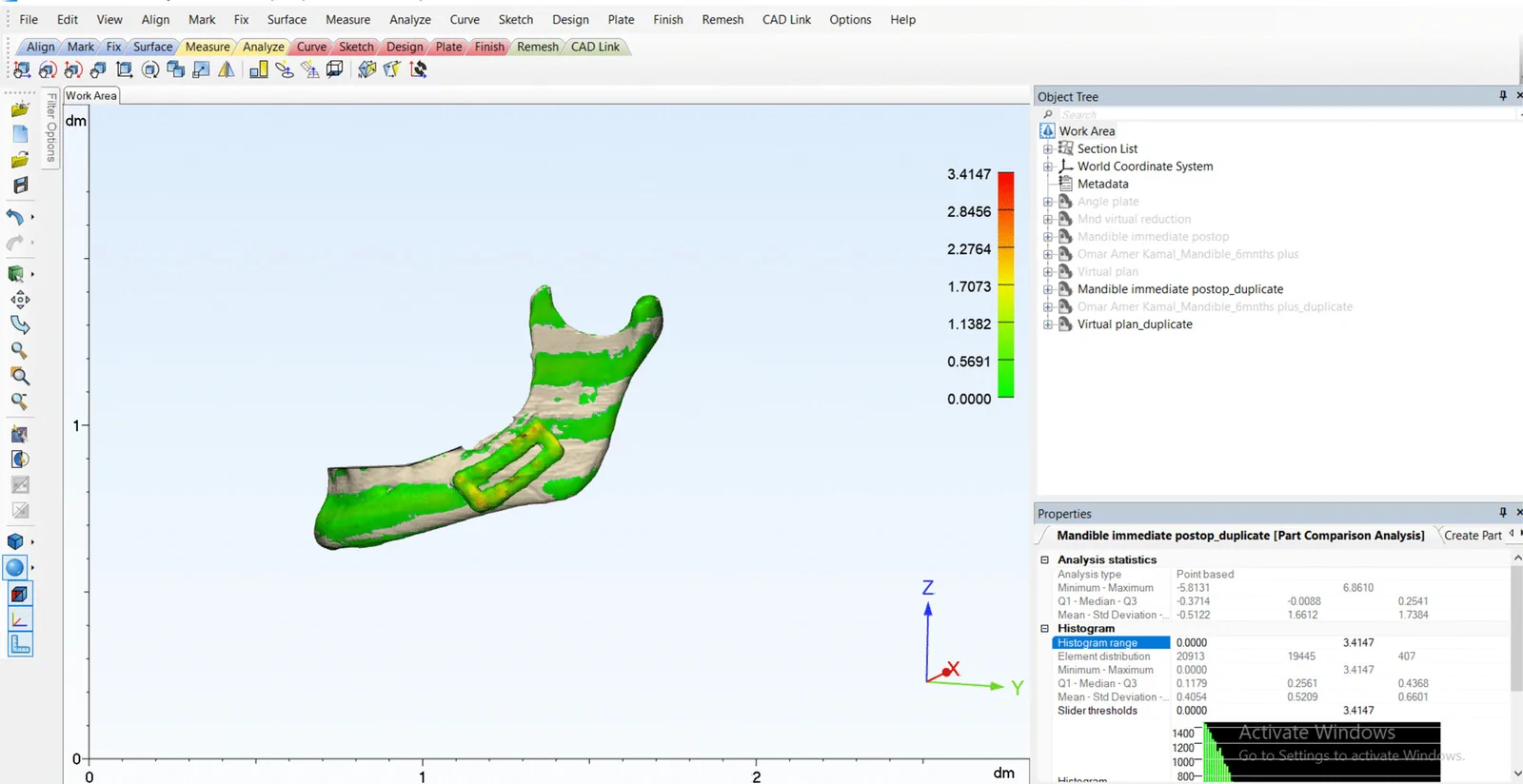
Superimposition of 3D virtual models of the preoperative virtual plan and postoperative surgically reduced and fixed mandible

For assessment of accuracy of VSP and CAD/CAM in guided reduction and stabilization of MAF using the fracture reduction/plate positioning PSSG and the customized three-dimensional (3D) miniplate, DICOM files from immediate and 6 months postoperative CT scans of *group I* were imported to the previously mentioned specialized viewer (Mimics21, Materialise, Leuven, Belgium) and segmented to obtain 3D virtual models of the reduced and fixed mandible at both time intervals, those, together with 3D virtual model of the preoperative virtually reduced mandible were then imported into the second software (3-Matic, Leuven, Belgium). 3D virtual models of 1 week and 6 months postoperative surgically reduced and fixed mandibles were then individually superimposed over the 3D virtual model of the preoperative virtually reduced mandible using the align tool (interactive translate, interactive rotate and global registration tools), followed by creating part comparison analysis from the analyze tool to obtain the amount of mean surface deviation between models in millimeters (Fig. 8).

### Statistical analysis

Categorical data were presented as frequencies and percentages. Demographic data were compared using the chi-squared test, with p-values estimated via Monte Carlo simulations. Occlusal discrepancy data were analyzed using a Firth-penalized generalized linear model with cluster-robust covariance matrix for inference. Continuous data, presented as mean ± standard deviation (SD), were examined for normality by visually inspecting the distributions and using the Shapiro–Wilk test, and showed no substantial deviation from normality. Bite force and surface deviation data were analyzed using mixed linear models, and the normality of the residuals was assessed as described earlier. Post hoc simple effects comparisons of estimated marginal means were performed using Welch’s and z-tests for the occlusal discrepancy model and F- and t-tests with Kenward-Roger approximation for the bite force and surface deviation models. Effect sizes were calculated and interpreted based on Cohen, J. (1992) and Vandekar, S. N., Tao, R., & Blume, J. D. (2020)[28, 29].The significance level was set at p < 0.05 for all tests. Statistical analysis was performed with R statistical analysis software version 4.5.2 for Windows[30].

## Results

This study was conducted on a total of 24 patients (9 males and 3 females in each group). Four patients were lost at follow-up visits (1 male and 1 female from *group I* and 2 males from *group II*). Accordingly, the data of only 20 patients was included for statistical analysis. The mean age of patients was 27.00 ± 3.41 years for *group I* and 26.83 ± 3.43 years for *group II*. The most prevalent etiology of fractures in both groups was road traffic accidents (80% and 70%) followed by interpersonal/domestic violence (10% and 20%) and falls (10% and 10%) for *groups I* and *II* respectively. The differences between groups in demographic and baseline characteristics were not statistically significant. Summary statistics of demographics and baseline characteristics are presented in Table 1.

**Table 1:** Summary statistics of demographics and baseline characteristics.CI: Confidence interval; ns: not significant

Parameter		Group I	Group II	p-value	Effect size (95% CI)**
Gender [n (%)]	Male	8 (80.00%)	7 (70.00%)	1 ns	0.00 (0.00 to 0.00)
	Female	2 (20.00%)	3 (30.00%)		
Age (years) (Mean ± SD)		27.00 ± 3.41	26.83 ± 3.43	0.906 ns	0.05 (−0.75 to 0.85)
Etiology of fracture [n (%)]	RTA	8 (80.00%)	7 (70.00%)	1 ns	0.13 (0.00 to 0.42)
	Interpersonal/domestic violence	1 (10.00%)	2 (20.00%)		
	Falls	1 (10.00%)	1 (10.00%)		

In both groups, bite force measurements increased significantly over time (p < 0.001) from 1 week to 6 months postoperatively, with a large effect size of 1. All pairwise comparisons were statistically significant. Bite forces in *group I* were significantly higher (p < 0.001) than those of *group II* at 1 week (20.48 ± 3.20N—13.24 ± 2.83N), 1 month (69.52 ± 3.42N—41.16 ± 3.02N) and 3 months (147.45 ± 3.00N—139.50 ± 3.44N), respectively, with large effect sizes (PES > 0.29). At six months, there was also a statistically significant difference (p = 0.003) between both groups (166.66 ± 2.8N—162.31 ± 3.19N respectively) with higher bite force in group I, however, the effect size was moderate (PES = 0.13). Difference in postoperative occlusion status was statistically insignificant between both groups across all time points. Comparisons and summary statistics of bite force (N) are presented in Table 2.

**Table 2:** Comparisons and summary statistics of bite force (N).PES: Partial Eta Squared; CI: Confidence interval; Values with different superscripts within the same vertical column are significantly different; * significant (*p* < 0.05)

	Bite force (N) (Mean ± SD)		p-value	PES (95% CI)
	Group I	Group II		
**1 week**	20.48 ± 3.20^D^	13.24 ± 2.83^D^	< 0.001*	**0.29 (0.12 to 0.45)**
**1 month**	69.52 ± 3.42^C^	41.16 ± 3.02^C^	< 0.001*	**0.86 (0.80 to 0.90)**
**3 months**	147.45 ± 3.00^B^	139.50 ± 3.44^B^	< 0.001*	**0.33 (0.16 to 0.48)**
**6 months**	166.66 ± 2.84^A^	162.31 ± 3.19^A^	0.003*	**0.13 (0.02 to 0.28)**
**p-value**	< 0.001*	< 0.001*		
**PES (95% CI)**	**1.00 (1.00 to 1.00)**	**1.00 (1.00 to 1.00)**		

In comparisons of the percentage of bite force gained in relation to the healthy control group, the percentages in *group I* were significantly higher (p-values ranging from 0.043 to < 0.001) than those in *group II* at 1 week (10.24 ± 1.6—6.62 ± 1.43), 1 month (34.85 ± 3.30—20.63 ± 2.25) and 3 months (73.82 ± 4.83—69.81 ± 4.10) respectively. However, after 1 week, the effect size was small (0.11); after 1 month, it was large (0.65); and after 3 months, it was moderate (0.13). After 6 months, the percentage was 83.43 ± 5.29 and 81.27 ± 5.51 for *group I* and *group II* respectively, yet the difference was not statistically significant (p = 0.219). Comparisons and summary statistics of bite force percentage in relation to healthy controls (%) are presented in Table 3.

**Table 3:** Comparisons and summary statistics of bite force percentage in relation to healthy controls (%). PES: Partial Eta Squared; CI: Confidence interval; * significant (p < 0.05); ns: not significant

Time	Bite force percentage in relation to healthy controls (%) (Mean ± SD)		p-value	PES (95% CI)
	Group I	Group II		
**1 week**	10.24 ± 1.68	6.62 ± 1.43	0.043*	**0.11 (0.00 to 0.32)**
**1 month**	34.85 ± 3.30	20.63 ± 2.25	< 0.001*	**0.65 (0.45 to 0.77)**
**3 months**	73.82 ± 4.83	69.81 ± 4.10	0.026*	**0.13 (0.00 to 0.34)**
**6 months**	83.43 ± 5.29	81.27 ± 5.51	**0.219 ns**	**0.04 (0.00 to 0.22)**

Both groups showed no statistically significant difference in occlusion status observed across time points (p = 0.907 and 1, for *group I* and *group II*, respectively). Similarly, all differences between both groups were not statistically significant (p = 0.283 and 0.601, respectively). At 1 week postoperatively, two cases in *group II* had a mild gap. While in *group I*, only 1 case had a mild gap at 1 month postoperatively. All other cases in both groups had satisfactory occlusion. Comparisons and summary statistics of occlusal discrepancy are presented in Table 4.

**Table 4:** Comparisons and summary statistics of occlusal discrepancy. RESI: Robust Effect Size Index; CI: Confidence interval; ns: not significant. *Occlusal status categorized based on the following scale: satisfactory (no gap), mildly deranged (1–2 mm gap), and deranged (> 2 mm gap) *as per Mujtaba et.al*[8]* and Vineeth *et al*.*[27]

Time	*Occlusal status	n (%)		p-value	RESI (95% CI)
		Group I	Group II		
**1 week**	**Satisfactory**	10 (100.00%)	8 (80.00%)	**0.283 ns**	**0.04 (0.00 to 0.34)**
	**Mild gap**	0 (0.00%)	2 (20.00%)		
	**Marked gap**	0 (0.00%)	0 (0.00%)		
**1 month**	**Satisfactory**	9 (90.00%)	8 (80.00%)	**0.601 ns**	**0.00 (0.00 to 0.27)**
	**Mild gap**	1 (10.00%)	2 (20.00%)		
	**Marked gap**	0 (0.00%)	0 (0.00%)		
**3 months**	**Satisfactory**	9 (90.00%)	8 (80.00%)	**0.601 ns**	**0.00 (0.00 to 0.27)**
	**Mild gap**	1 (10.00%)	2 (20.00%)		
	**Marked gap**	0 (0.00%)	0 (0.00%)		
**6 months**	**Satisfactory**	9 (90.00%)	8 (80.00%)	**0.601 ns**	**0.00 (0.00 to 0.27)**
	**Mild gap**	1 (10.00%)	2 (20.00%)		
	**Marked gap**	0 (0.00%)	0 (0.00%)		
**p-value**		**0.907 ns**	**1 ns**		
**RESI (95% CI)**		**0.00 (0.00 to 0.19)**	**0.00 (0.00 to 0.00)**		

Superimposition of 3D virtual models of the surgically reduced mandible at 1 week and 6 months over 3D virtual models of the preoperative virtual reduction plan revealed mean surface deviations of 0.36 ± 0.09 mm and 0.45 ± 0.08 mm, respectively.

## Discussion

When managing mandibular fractures, the ultimate goal is the restoration of the patient’s masticatory function and facial esthetics. To reach this goal, anatomical reduction of the fractured segments to the pre-trauma status of the mandible and their stabilization until complete bone healing is paramount. It is beyond doubt that digitization and customization are now widespread within the surgical field, with claims that many of the drawbacks of the traditional methods of management of MAF can be overcome with incorporation of advanced technologies. Today, with the help of specialized softwares and CAD/CAM technologies, surgeons can preoperatively tailor a complete surgical plan virtually and simultaneously design and produce customized, patient specific devices to enhance surgical outcomes, reduce the rate of complications and expedite restoration of function[19, 31].In this study, we set out to investigate the accuracy of VSP and CAD/CAM in creating a fracture reduction/plate positioning PSSG and a customized 3D miniplate to achieve anatomical reduction and stabilization in MAF via a more esthetic, less complication-laden intraoral access, and to evaluate efficiency of customized 3D miniplates in reestablishment of masticatory function.

PSSGs are extensively used in surgery. Among several uses, PSSGs can be designed to act as drill guides for predestined screw positions in different surgical procedures [19, 23]. In our study, we designed a PSSG with drill guides coinciding with the screw holes of customized 3D miniplates for MAF stabilization. The resin PSSG was designed to “snap on” and securely fit through an intraoral approach onto the sigmoid notch and the inferior and posterior borders of the mandible to ensure its stability during drilling through the drill guides and guarantee that screw holes are drilled in the proper preplanned position. We designed the PSSG as a two-piece guide to allow intraoral visualization of the reduced segments during drilling; it was also more convenient to use the two-piece guide for easier placement through the intraoral incision rather than a bulkier one-piece guide. Intraoperatively, we were able to place the PSSG into its predestined position in accordance with the virtual preoperative plan. Yadav et al. reported unsuccessful placement of non-guided 3D miniplates due to poor visibility or surgeon misjudgment [15]. In contrast, Ramanathan et al. demonstrated the effectiveness of surgical guides in accurate fracture reduction in MAF [13]. In their study, El-Gengehi and Seif [32] used a CAD/CAM software to perform virtual anatomical reduction of fractured mandibular segments and concluded that virtually designed customized surgical reduction guides aid in obtaining accurate anatomical reduction of displaced mandibular fractured segments. Similarly, the feasibility and accuracy of virtual planning and CAD/CAM patient specific osteosynthesis plates and drilling guides were reported for management of tibial[23] and acetabular[33] fractures. The results of our study are consistent with these findings. It is worth mentioning that when designing screw holes of the 3D miniplates, the merits of being able to visualize the position of the vital structures such as the inferior alveolar canal and roots of teeth in the vicinity of the line of fracture during virtual planning allowed us to plan the exact position of fixation screws without causing injury to these structures.

In our findings, there was no statistical difference between both groups at all follow-up intervals regarding occlusion status, similar findings were reported by Mujtaba et al. [8] in a comparison of postoperative occlusion between 3D and 2D miniplates in management of isolated MAFs. On the other hand, despite improvement over time, bite force measurements showed a significant difference at all follow-up intervals between both groups in favor of 3D miniplate fixation.

Champy's single miniplate fixation along the ideal line of osteosynthesis has been strongly recommended by many authors for the management of mandibular angle fractures [4, 9, 34, 35]. Since clinical evidence consistently shows that absolute rigid fixation is not a prerequisite for predictable fracture healing at the mandibular angle[1, 6], the semi-rigid stabilization provided by Champy's technique is considered biomechanically sufficient for uncomplicated, non-comminuted fractures, especially as the ptreygo-masseteric muscular sling provides additional passive stabilization that is not accounted for in laboratory models [5, 35]. Meta-analytic evidence has also shown that single-plate fixation reduces the risk of postoperative complications by 37% compared with two-plate fixation [34]. Such data support the fact that the clinical performance of Champy’s technique should not be necessarily undermined by its theoretical biomechanical limitations. Its clinical validity relies on it being a less invasive approach that offers sufficient stability for most uncomplicated cases, with lower complication rates such as infection, wound dehiscence, scarring, and facial nerve injury, as well as less hardware failure, shorter operative time, and reduced cost [3, 16, 36, 37]. Nevertheless, others stated disadvantages to Champy’s technique such as splaying of inferior border when occlusal loading forces are applied near the fracture line, plate fracture due to fatigue stresses induced by bending for adaptation and cyclic masticatory load, wound dehiscence and plate exposure, and postoperative infection [6, 8, 36, 38].

On the other hand, 3D miniplates utilize the concept of the two-plate fixation method along tension and compression zones in a single plate configuration with the added advantage of increased biomechanical stability by counteracting torsional forces via vertical bars while maintaining malleability and a low profile [2, 8, 11]. The stability of those plates depends mostly on their format rather than their thickness [39]. Recently, there is an increase in literature reports on customized patient specific plates, Du et al. [20] reported that high quality patient specific plates can be designed, fabricated and successfully used in craniofacial reconstruction with geometrical accuracy, optimal biomechanical properties, and a short recovery time. The customized 3D miniplates we used in this study were custom-fabricated in 1 mm thickness from Grade 5 titanium of high mechanical properties[40, 41]. They were designed to combine the concepts of two-plate fixation and 3D design, while adopting the convenience of placement via an intraoral incision and avoiding the setbacks of extraoral approaches. Those 3D miniplates were fixed superiorly with monocortical screws and at the inferior border with bicortical screws for more stability. In their study, Pfister et al. [42] concluded the feasibility of treatment of MAFs using a 1.0 mm thick, square shaped 3D plate system, while Squier et al. [43] recommended the use of 3D plates over single plates based on their findings of superior tolerance of 3D plates to load as compared to single plate fixation in MAFs. Alkan et al. [44] demonstrated that 3D plates had greater resistance to compression loads than Champy’s technique. Clinically, several studies stated better initial interfragmentary stability [27, 45] and less likelihood of postoperative complications using 3D miniplates[46]. AlMoraissi et al., in their systematic review comparing clinical outcomes of standard and 3D miniplate fixation in MAFs, stated that 3D miniplate fixation reduced the risk of postoperative complications and provided greater interfragmentary stability compared to standard miniplates[34].

The major determinants of masticatory function are; the range of mandibular motion, maximum occlusal forces, and the activity of the masticatory muscles, therefore, bite force measurements are an excellent criterion for the assessment of masticatory efficiency[18].In their comparative study for assessment of the standard and 3D miniplates in managing isolated MAFs, Saxena et al.[47] reported improvement of bite force at 1 week, 1 months and 3 months follow-up intervals in both groups with higher mean bite force values in patients treated with 3D plates. Similar findings were reported by Kinra et al.[48] with a maximum follow-up period of two months. Our findings demonstrate similar results of bite force suggesting that patients in *group I* were able to generate and tolerate greater masticatory loads earlier in the healing period. It is likely, that the combination of; 3D design of the miniplate, the superior material quality from which it was fabricated (grade 5 titanium) and the method of fixation (superiorly with monocortical screws and at the inferior border with bicortical screws), contributed to the higher bite force observed in *group I*. However, this assumption needs to be supplemented by biomechanical analysis and further clinical studies on larger sample sizes to support or refute this explanation, it remains interpretive in nature and must be regarded with caution . The bite force data in this study merely serve as a functional rehabilitation outcome reflecting the degree to which each fixation method allowed early, progressive restoration of masticatory loading.

Finally, to assess the accuracy of VSP, virtual 3D models of the mandible from immediate and 6 months postoperative CT scans of *group I* patients were compared to those of the virtual preoperative plan by digital superimposition. Our findings revealed minimal amounts of mean surface deviation of 0.36 ± 0.09 mm and 0.45 ± 0.08 mm between virtual 3D models of the virtual surgical plan and those of postoperative CT scans at 1 week and 6 months postoperative follow-up intervals, respectively, demonstrating a considerable level of accuracy and negligible discrepancy. In their systematic review on VSP in orthognathic surgery, Alkhayer et al.[49] concluded that differences less than 2 mm between the virtual plan and postoperative outcomes are considered accurate and acceptable. While Gaber et al.[50], reported that assessment of accuracy of VSP in computer-guided mandibular fracture reduction using superimposition, showed mean bony and dental deviations of 0.09 ± 0.29 mm and 0.05 ± 0.16 mm respectively, as compared to the control group reaching 0.70 ± 0.33 mm and 0.56 ± 0.32 mm respectively, they stated that deviations in both groups of their study as negligible compared to the 2 mm threshold. The findings of our study fall well within this reported threshold. It is worth mentioning that Nilsson et al.[51] also point out that superimposition-based measurements can overestimate the actual clinical discrepancy, as a minimal rotation at the fracture site propagates into larger deviations when measured at peripheral landmarks. The values of our study therefore likely represent an even smaller discrepancy at the fracture site itself.

Restoration of masticatory function is one of the primary objectives in the management of MAFs which requires precise anatomical reduction of the fractured segments to their pre-trauma position thus restoring the pre-trauma occlusal relationships, followed by their stabilization in that position to ensure bone-to-bone contact minimizing the interfragmentary gap in order to optimize the osseous healing environment [7, 12]. Accordingly, mal-aligned fracture segments compromise bone healing and predispose to a range of postoperative complications [10, 12, 34], these include delayed union, malunion, or non-union, functional impairment, increase susceptibility to infection, hardware failure, and the need for revision surgery [10, 34, 36]. At the dental level, segment malalignment disrupts the pre-trauma occlusion, resulting in postoperative malocclusion that impairs masticatory efficiency and may impose abnormal loading on the temporomandibular joints [7, 10, 36]. As we mentioned earlier, bite force measurements serve as an indicator of masticatory functional recovery following surgical treatment. In their study, Sybil et al. [52]reported bite force below 65% of normal values at nine weeks postoperatively following miniplate osteosynthesis, while other literature stated bite force recovery reaching only 31% of normal values at one week postoperatively and approximately 58% by the sixth week [48, 53].

In this context, the clinical significance of the minimal amount of deviation of superimposition in this study is reflected on the clinical outcomes of occlusal status where satisfactory occlusion was observed across all follow-up intervals in *group I* as well as early reestablishment of masticatory function and a progressive gain in bite force of 10.24 ± 1.68% at 1 week postoperative and 34.85 ± 3.30% at 1 month reaching 73.82% ± 4.83 and 83.43% ± 5.29 at 3 and 6 months postoperative, respectively in relation to bite force values of healthy controls.

A key advantage of VSP and CAD/CAM technology is the ability to design and fabricate customized PSSDs such as PSSGs and customized miniplates that precisely conform and configurate anatomically to each patient's mandibular morphology. In contrast, conventional stock miniplates require intraoperative manual bending and adaptation to obtain passive accurate fit to the bone surface, which is technique-sensitive and induces internal stresses within miniplates. An ill-fitting or misfitting plate, where the plate does not conform accurately to the bone surface generates unwanted stresses at the bone-plate interface upon screw tightening, which can displace the reduced fragments and compromise the anatomical reduction. This may manifest as malocclusion, malunion, or non-union, and can further predispose to hardware failure, wound dehiscence, plate exposure and infection as well as prolonged surgical operative time [6, 8, 10, 36, 37] “

In this study, intraoperatively, passive placement and fixation of the customized 3D miniplate was obtained, which, in our opinion, clinically supports the observed minimal discrepancy by superimposition and the level of accuracy of VSP, along with the observation of being able to place the PSSG intraoperatively into its predestined position as the virtual preoperative plan. Additionally, upon examination of postoperative CT scans of *group I* patients, no fixation screws were found interfering with the inferior alveolar canal or the roots of any teeth within the vicinity of the fracture. In their study, Kreutzer et al. [54]investigated the accuracy of VSP for free fibular flap fixation in mandibular reconstruction and observed high levels of accuracy, others also reported on the effectiveness of VSP and CAD/CAM surgical osteotomy- and drill-guides in orthognathic and reconstructive surgeries [55, 56]. Also, in their review, Moolenaar et al.[57] stated that current computer-assisted planning approaches are feasible for use in clinical practice and have been shown to improve clinical outcomes.

Despite their merits and promising outcomes, VSP and CAD/CAM technologies retain limitations including the availability of CT scanning, planning softwares, and facilities capable of fabricating customized devices, along with the added cost they entail [19, 31].These factors may limit the wider adoption of such techniques, particularly in medical care facilities with limited resources. Another limitation to be taken into consideration is the learning curve required to achieve efficient VSP and that to accurately execute the virtual preoperative plan intraoperatively, which may represent a practical barrier in acute trauma settings. Studies have reported on the complexity of some VSP platforms [22, 51], and others reported planning durations ranging from 22 to 258 min and multi-step engineer-surgeon correspondence potentially introducing delays of several days [51, 57]. Kim et al.[22] also stated that the accurate intraoperative transfer of the virtual plan to the surgical field remains technically demanding even in experienced hands.

## Conclusion

VSP revealed a minimal amount of discrepancy between 3D virtual models of the surgically reduced mandible and the 3D virtual models of the preoperative virtual reduction plan denoting a high level of accuracy of VSP. An anatomically oriented, bone-borne fracture reduction/plate positioning PSSG aided significantly in customized 3D miniplate positioning and fracture reduction. Fractures stabilized with a customized 3D miniplate were associated with higher bite forces when compared to single miniplate fixation, however, this must be interpreted with caution. We recommend that future studies be conducted on a larger sample size.

## Clinical relevance

Based on the results of this study, we believe that utilizing VSP for guided ORIF of MAF using customized 3D miniplate seems to be a promising method, allowing predictable plate positioning and fixation through an intraoral approach as well as early reestablishment of masticatory function.

## Data Availability

The datasets used and analyzed during the current study are available from the corresponding author upon reasonable request.

## Footnotes

1 Hibiotic 1 gm, Amoun Pharmaceuticals, El Obour city, Cairo, Egypt.

2 Flagyl 500 mg, Sanofi-Aventis, SAE, El-Sawah, El-Amirya, Egypt.

3 Brufen 600 mg, Abbott Pharmaceuticals, Kahira Pharm. & Chem. Ind. Co., Egypt.

4 Hexitol mouthwash, Arab Drug Company, Cairo, Egypt.
